# A Robust Cubature Kalman Filter with Abnormal Observations Identification Using the Mahalanobis Distance Criterion for Vehicular INS/GNSS Integration

**DOI:** 10.3390/s19235149

**Published:** 2019-11-25

**Authors:** Bingbing Gao, Gaoge Hu, Xinhe Zhu, Yongmin Zhong

**Affiliations:** 1 School of Automation, Northwestern Polytechnical University, Xi’an 710072, China; hugaoge1111@126.com; 2 School of Engineering, RMIT University, Bundoora, VIC 3083, Australia; xinhe.zhu@rmit.edu.au (X.Z.); yongmin.zhong@rmit.edu.au (Y.Z.)

**Keywords:** vehicular navigation, INS/GNSS integration, cubature Kalman filter, abnormal observations identification, Mahalanobis distance criterion

## Abstract

INS/GNSS (inertial navigation system/global navigation satellite system) integration is a promising solution of vehicle navigation for intelligent transportation systems. However, the observation of GNSS inevitably involves uncertainty due to the vulnerability to signal blockage in many urban/suburban areas, leading to the degraded navigation performance for INS/GNSS integration. This paper develops a novel robust CKF with scaling factor by combining the emerging cubature Kalman filter (CKF) with the concept of Mahalanobis distance criterion to address the above problem involved in nonlinear INS/GNSS integration. It establishes a theory of abnormal observations identification using the Mahalanobis distance criterion. Subsequently, a robust factor (scaling factor), which is calculated via the Mahalanobis distance criterion, is introduced into the standard CKF to inflate the observation noise covariance, resulting in a decreased filtering gain in the presence of abnormal observations. The proposed robust CKF can effectively resist the influence of abnormal observations on navigation solution and thus improves the robustness of CKF for vehicular INS/GNSS integration. Simulation and experimental results have demonstrated the effectiveness of the proposed robust CKF for vehicular navigation with INS/GNSS integration.

## 1. Introduction

The Internet of Things (IoT) is a burgeoning concept of connected objects operating together to exchange information with each other [1]. As a prototypical IoT application, intelligent transportation has attracted great research interest during the past several decades [2,3]. Currently, global navigation satellite system (GNSS)-based navigation technology plays an important role in intelligent transportation systems to provide location information for vehicles. GNSS can output a seamless positioning solution for vehicles in an open sky environment with good satellite visibility [4,5]. However, it is difficult for GNSS-alone positioning to satisfy the stringent requirements of vehicle positioning due to the vulnerability to signal blockage in many urban/suburban areas [5,6,7]. Moreover, due to their inherent low power, GNSS signals are susceptible to interference, leading to the problems of deliberate spoofing and jamming [6,7,8].

To address the limitations of GNSS, a commonly used strategy is to augment GNSS with a complementary navigation system which is insusceptible to external signal interruptions or interferences [9,10,11]. For this purpose, the inertial navigation system (INS), a self-contained system that is immune to jamming/interference, is often integrated with GNSS [11,12,13]. GNSS and INS can complement each other in terms of error characteristics: GNSS has good long-term accuracy; In contrast, INS operates continuously and provides navigation solutions with good short-term accuracy [14], while it suffers from accuracy degradation over time due to the drifts of inertial measurement units [15]. Thus, their complementary features make INS/GNSS integration become a promising solution for vehicle navigation, especially when the observability of GNSS signals is quite poor.

Data fusion processing is the most important procedure to solve for the navigation solution of INS/GNSS integration [9,13]. The Kalman filter (KF) and its variants have been widely applied in INS/GNSS integration [16]. However, they are only suitable for linear systems due to their theoretical limitations, while the system model of INS/GNSS integration is strongly nonlinear due to vehicle maneuvering [5,17]. The extended Kalman filter (EKF) and unscented Kalman filter (UKF) are the commonly used filtering strategies for nonlinear systems [18,19,20]. UKF uses a set of deterministically selected sigma points to approximate the probability distribution of system state and further propagates them through the nonlinear system model, leading to much higher-order approximation accuracy than EKF [19,20]. However, UKF is considered to be unstable due to the occurrence of negative weights, especially for high-dimensional (more than 3) nonlinear systems [21,22].

The cubature Kalman filter (CKF) is an emerging nonlinear filtering technology [22,23]. CKF exploits a third-degree spherical-radical cubature rule to calculate the involved Gaussian weighted integrals, leading to improved accuracy [23,24]. Compared to EKF and UKF, CKF has several advantages [21,22,23,24]: (i) since the Gaussian-weighted integrals are numerically calculated through third-degree spherical-radial cubature, CKF has better approximation accuracy than EKF and UKF; (ii) CKF does not require the nonlinear equation to be differential, while EKF does; (iii) CKF does not involve any free parameter, and thus it has better numerical stability in comparison to UKF; (iv) Under the same order of computational complexity as EKF and UKF, CKF can achieve more accurate and stable estimation results for high dimension nonlinear systems. However, similar as EKF and UKF, the implementation of CKF requires the system model to be pre-defined exactly. If the system model involves errors, the performance of CKF will be deteriorated [25,26]. For INS/GNSS integrated vehicle navigation, the precision of the dynamic model can be ensured by using laser or optical inertial measurement units of ultra-high accuracy [27]. Nevertheless, the observation of GNSS can be degraded in the environments such as urban canyons, tunnels, and foliage conditions (tree canopies), resulting in the failure to provide continuous and superior navigation solutions [1,3]. Therefore, the study of a new CKF with the robustness against abnormal observations appears to be particularly important for vehicular INS/GNSS integration.

Research efforts have been dedicated to improving the robustness of the standard CKF against abnormal observations [17,28,29,30,31,32]. According to the concept of Sage-Husa noise statistics estimation method, Ding and Xiao derived an adaptive CKF with observation noise statistics estimator to improve the robustness of CKF against abnormal observations [28]. Nevertheless, with this method, the “rank deficient” issue may occur in the estimation of noise statistics for high-dimensional nonlinear systems, leading to unstable filtering outputs [33]. Liu et al. proposed a maximum correntropy square-root CKF to address the problem of non-Gausssian observation noise involved in INS/GNSS integration [17]. However, the construction of the estimation error covariance matrix is not based on theoretical analysis [34], making the improvement of this method questionable. Zhang et al. developed an H-infinity strategy based robust CKF by minimizing the estimation error in the worst case [29]. However, it may break down in the presence of randomly occurring abnormal observations [33,35]. The combination of the M-estimation theory with CKF was also studied to resist the influence of abnormal observations on dynamic state estimation [30,31]. However, this method achieves the robustness at the cost of reducing the accuracy of the nonlinear transformation itself [33,36]. Zhao et al. designed a robust strong tracking CKF and developed a noise statistic estimator based on the principle of maximum a posterior to overcome the model uncertainty caused by abnormal observations [32]. However, the forgetting factors used in this filter have to be determined empirically for the case of time-variant noises.

Mahalanobis distance is a criterion to identify outliers for multivariate data in statistics [35,37]. Chang established a robust KF by combining the Mahalanobis distance criterion with the linear KF to improve the filtering robustness [35]. However, as stated previously, due to the theoretical limitation, this method can only be used for a linear system, unsuitable for a nonlinear system such as INS/GNSS integration. To the best of our knowledge, there has been very limited research on combining the Mahalanobis distance criterion with the nonlinear CKF for nonlinear INS/GNSS integration.

This paper presents a novel robust CKF with scaling factor to improve the robustness of nonlinear vehicular INS/GNSS integration. The proposed method establishes novel Mahalanobis distance criterion theories to improve the robustness of the standard CKF. It employs the concept of the Mahalanobis distance criterion to identify abnormal observations involved in nonlinear INS/GNSS integration. Based on this, a robust factor (scaling factor) is developed via the Mahalanobis distance criterion and is introduced into the standard CKF to inflate the observation noise covariance, thus decreasing the filtering gain to resist the influence of abnormal observations on dynamic state estimate. The proposed robust CKF can accommodate the influence of abnormal observations on navigation solution, resulting in the improved robustness for vehicular INS/GNSS integration. Simulations and practical experiments have been conducted to comprehensively evaluate the performance of the proposed robust CKF for INS/GNSS integrated vehicle navigation.

## 2. System Model of Vehicular INS/GNSS Integration

Vehicular INS/GNSS integration allows us to completely exploit the individual advantages of INS and GNSS by using high-precision GNSS position and velocity to correct the INS drift errors, leading to a feasible solution to enhance the accuracy of vehicle navigation.

### 2.1. Dynamic Model

Vehicular INS/GNSS integration uses the standard inertial navigation equations as the dynamic model. Define the inertial frame as *i*, the body frame as *b*, and the Earth frame as *e*. The *E-N-U* (*East-North-Up*) geography frame (*g*) is selected as the navigation frame (*n*). Then, the standard inertial navigation equations using quaternion parameterization are given by [5,18]:

(1) $${\dot{q}}_{b}^{n}=\frac{1}{2}q_{b}^{n}⊗\omega_{nb}^{b}$$

(2) $${\dot{v}}^{n}=C(q_{b}^{n})f^{b}-(2\omega_{ie}^{n}+\omega_{en}^{n})\times v^{n}+g^{n}$$

(3) $${\dot{p}}^{n}=Mv^{n}$$

In (1), $q_{b}^{n}$ is the attitude quaternion from the *b*-frame to *n*-frame for the vehicle, ⊗ denotes the quaternion multiplication, and $\omega_{nb}^{b}$ is the body angular rate with respect to the *n*-frame and is described as:

(4) $$\omega_{nb}^{b}=\omega_{ib}^{b}-C(q_{b}^{n})(\omega_{ie}^{n}+\omega_{en}^{n})$$

where $\omega_{ib}^{b}$ is the body angular rate measured by gyroscopes in the *b*-frame; $\omega_{ie}^{n}$ is the earth rotation rate in the *i*-frame; $\omega_{en}^{n}$ is the angular rate of the *n*-frame with respect to the *e*-frame; and $C(q_{b}^{n})$ denotes the attitude matrix corresponding to the attitude quaternion $q_{b}^{n}$.

In (2) and (3), $v^{n}={\left[v_{E}^{n},v_{N}^{n},v_{U}^{n}\right]}^{T}$ represents the vehicular velocity relative to the Earth; $g^{n}$ is the gravity vector; $f^{b}$ denotes the specific force measured by accelerometers in the *b*-frame, $p^{n}={\left[\lambda ,L,h\right]}^{T}$ is the vehicle position in longitude, latitude and altitude; and M is represented as:

(5) $$M=\left[\begin{array}{ccc} \frac{1}{(R_{N}+h)\cos L} & 0 & 0\\ 0 & \frac{1}{R_{M}+h} & 0\\ 0 & 0 & 1 \end{array}\right]$$

where $R_{M}$ and $R_{N}$ are the radii of curvature in meridian and prime vertical, respectively.

The measurement model of the gyro is defined as:

(6) $${\tilde{\omega }}_{ib}^{b}=\omega_{ib}^{b}+\varepsilon_{}^{b}+\eta_{gv}$$

(7) $${\dot{\varepsilon }}_{}^{b}=\eta_{gu}$$

where $\varepsilon_{}^{b}$ is the gyro bias; and $\eta_{gv}$ and $\eta_{gu}$ obey the zero-mean Gaussian white noise processes with spectral densities.

Similarly, the measurement model of the accelerometer is described as:

(8) $${\tilde{f}}_{}^{b}=f_{}^{b}+\nabla^{b}+\eta_{av}$$

(9) $${\dot{\nabla }}^{b}=\eta_{au}$$

where $\nabla^{b}$ is the accelerometer bias; and $\eta_{av}$ and $\eta_{au}$ are the zero-mean Gaussian white noise processes with spectral densities.

It should be noted that the attitude quaternion in (1) must obey a normalization constraint, which may be violated due to the inherent averaging operation of CKF. This problem can be addressed by utilizing three-dimensional Generalized Rodrigues Parameters (GRPs) to represent the quaternion error vector and update it via quaternion multiplication. Based on this, the normalization constraint can be maintained [5,38].

Represent the attitude error quaternion as $\delta q={\left[\delta q_{0},\delta u^{T}\right]}^{T}$, where δu is the vector part of the quaternion δq. The GRP corresponding to the error quaternion δq is defined as:

(10) $$\delta R=l\frac{\delta u}{s+\delta q_{0}}$$

where *s* is a constant within [0,1], and *l* is a scale factor [5].

Denote the system state x(t) as:

(11) $$x(t)={\left[{\left(\delta R\right)}^{T},{\left(v_{}^{n}\right)}^{T},{\left(p_{}^{n}\right)}^{T},{\left(\varepsilon_{}^{b}\right)}^{T},{\left(\nabla_{}^{b}\right)}^{T}\right]}^{T}$$

The dynamic model for vehicular INS/GNSS integration can be described as:

(12) $$\dot{x}(t)=\bar{f}(x(t))+w(t)$$

where $\bar{f}\left(\cdot \right)$ is the nonlinear function describing the dynamic model in continuous form, and w(t) is the process noise vector.

By discretizing (12) via the improved Euler method [39], the discrete-time dynamic model of vehicular INS/GNSS integration is obtained by:

(13) $$x_{k}=f\left(x_{k-1}\right)+w_{k}$$

where f(⋅) is a nonlinear function describing the dynamic model in discrete form; and w(k) is the discrete-time process noise vector.

### 2.2. Observation Model

The observation model for vehicular INS/GNSS integration is described as [8]:

(14) $$z_{k}=H_{k}x_{k}+v_{k}$$

where $H_{k}=\left[\begin{array}{ccc} 0_{6\times 3} & I_{6\times 6} & 0_{6\times 6} \end{array}\right]$, $v_{k}$ is the observation noise vector, and the observation vector $z_{k}$ is selected as:

(15) $$z_{k}={\left[v_{E},v_{N},v_{U},\lambda_{G},L_{G},h_{G}\right]}^{T}$$

where $\left(v_{E},v_{N},v_{U}\right)$ is the velocity vector output by the GNSS receiver and $\left(\lambda_{G},L_{G},h_{G}\right)$ is the position vector given by the GNSS receiver.

## 3. Robust Cubature Kalman Filter Based on the Mahalanobis Distance Criterion

In this section, a new approach of abnormal observation identification is established using the concept of the Mahalanobis distance criterion. Based on this, a novel robust CKF with scaling factor is further developed to resist the influence of abnormal observations on system state estimation.

### 3.1. Standard Cubature Kalman Filter

To show the derivation of the proposed robust CKF more clearly, the concept of the standard CKF is briefly reviewed at first. Consider the nonlinear discrete-time system consisting of (13) and (14)

(16) $$\{\begin{array}{c} x_{k}=f\left(x_{k-1}\right)+w_{k}\\ z_{k}=H_{k}x_{k}+v_{k} \end{array}$$

where $x_{k}\in R^{n}$ and $z_{k}\in R^{m}$ denote the state and measurement at time k of the dynamic system; $w_{k}$ and $v_{k}$ are the uncorrelated zero-mean Gaussian white noises with covariances $E\left[w_{k}w_{k}^{T}\right]=Q$ and $E\left[v_{k}v_{k}^{T}\right]=R$; and f(⋅) is the nonlinear function describing the dynamic model and $H_{k}$ is the observation matrix.

CKF is a deterministic sampling filter based on the third-degree spherical-radical cubature rule for nonlinear systems. The computational process of the standard CKF for the nonlinear system described by (16) can be summarized as:

*Step 1:* Initialization.

Initialize the state estimate ${\hat{x}}_{0}$ and its error covariance $P_{0}$ with:

(17) $$\left\{ \begin{array}{l} {\hat{x}}_{0}=E[x_{0}]\\ P_{0}=E[(x_{0}-{\hat{x}}_{0}){\left(x_{0}-{\hat{x}}_{0}\right)}^{T}] \end{array}\right.$$

*Step 2:* Time update.

Assume state estimate ${\hat{x}}_{k-1}$ and its error covariance matrix $P_{k-1}$ are given. The cubature points can be calculated by:

(18) $$P_{k-1}=S_{k-1}S_{k-1}^{T}$$

(19) $$\chi_{i,k-1}^{}={\hat{x}}_{k-1}+S_{k-1}\xi_{i}$$

where $S_{k-1}$ is computed by the Cholesky decomposition of $P_{k-1}$; and $\xi_{i}$ is selected as:

(20) $$\xi_{i}=\left\{ \begin{array}{ll} \sqrt{n}e_{i}, & i=1,2,\cdots ,n\\ -\sqrt{n}e_{i-n}, & i=n+1,n+2,\cdots 2n \end{array}\right.$$

where $e_{i}$ denotes the *i*th column vector of the n×n identity matrix.

Each of the cubature points is transformed through the dynamic model to yield a new set of points

(21) $$\chi_{i,k/k-1}^{}=f(\chi_{i,k-1}^{})\left(i=1,2\cdot \cdot \cdot ,2n\right)$$

Subsequently, the predicted state mean and covariance are calculated as:

(22) $${\hat{x}}_{k/k-1}=\frac{1}{2n}\sum_{i=1}^{2n}\chi_{i,k/k-1}^{}$$

(23) $$P_{k/k-1}=\frac{1}{2n}\sum_{i=1}^{2n}\chi_{i,k/k-1}^{}\chi_{i,k/k-1}^{T}-{\hat{x}}_{k/k-1}{\hat{x}}_{k/k-1}^{T}+Q$$

*Step 3:* Observation update.

Since the observation model is a linear model, the observation update process can be conducted in a similar way as KF.

(24) $${\hat{z}}_{k/k-1}=H_{k}{\hat{x}}_{k/k-1}$$

(25) $$P_{{\hat{z}}_{k/k-1}}=H_{k}P_{k/k-1}H_{k}^{T}+R$$

(26) $$P_{{\hat{x}}_{k/k-1}{\hat{z}}_{k/k-1}}=P_{k/k-1}H_{k}^{T}$$

(27) $$K_{k}=P_{{\hat{x}}_{k/k-1}{\hat{z}}_{k/k-1}}P_{{\hat{z}}_{k/k-1}}^{-1}$$

(28) $${\hat{x}}_{k}={\hat{x}}_{k/k-1}+K_{k}(z_{k}-{\hat{z}}_{k/k-1})$$

(29) $$P_{k}=P_{k/k-1}-K_{k}P_{{\hat{z}}_{k/k-1}}K_{k}^{T}$$

*Step 4:* Repeat Steps 2 and 3 for the process of all the samples.

It is obvious that if the system model (16) involves abnormal observations, it will directly decrease the estimation accuracy of the system state from (28), deteriorating the navigation performance for vehicular INS/GNSS integration. Therefore, it is necessary to establish a way to resist the influence of abnormal observations on the navigation solution.

### 3.2. Robust Cubature Kalman Filter Based on Mahalanobis Distance Criterion

#### 3.2.1. Mahalanobis Distance Criterion for Abnormal Observation Identification

The Mahalanobis distance is a criterion to detect outliers for multivariate data in statistics. For a multi-dimensional vector $x={\left(x_{1},x_{2},\cdots ,x_{p}\right)}^{T}$ with the mean $\mu ={\left(\mu_{1},\mu_{2},\cdots ,\mu_{p}\right)}^{T}$ and the covariance Σ, its Mahalanobis distance is defined as [16,39]

(30) $$D(x)=\sqrt{{\left(x-\mu \right)}^{T}\Sigma^{-1}(x-\mu )}$$

To identify abnormal observations involved in vehicular INS/GNSS integration using the Mahalanobis distance criterion, we first denote the innovation vector as:

(31) $${\tilde{z}}_{k}=z_{k}-{\hat{z}}_{k/k-1}$$

For a Gaussian system without abnormal observations, ${\tilde{z}}_{k}$ should obey the zero-mean Gaussian distribution with the covariance:

(32) $$P_{{\tilde{z}}_{k/k-1}}=H_{k}P_{k/k-1}H_{k}^{T}+R$$

Following the Mahalanobis distance’s definition given in (30), we have:

(33) $$\gamma_{k}^{}=D^{2}({\tilde{z}}_{k}^{})={\tilde{z}}_{k}^{T}P_{{\tilde{z}}_{k/k-1}^{}}^{-1}{\tilde{z}}_{k}^{}$$

It can be obtained from statistical analysis that the square of the Mahalanobis distance of the innovation vector should obey the chi-square distribution with *m* degrees of freedom [35,39], i.e.,

(34) $$\gamma_{k}^{}=D^{2}({\tilde{z}}_{k}^{})∼\chi_{m}^{2}$$

According to the hypothesis testing theory, for a given significance level α(0<α<1), there has a threshold $\chi_{m,\alpha }^{2}$ to make the following hold:

(35) $$P\{\gamma_{k}^{}>\chi_{m,\alpha }^{2}\}=\alpha$$

where P(⋅) represents the probability of a random event.

Thus, we can establish the following criterion to identify abnormal observation:

(36) $$\left\{ \begin{array}{l} H_{0}:\gamma_{k}^{}\leq \chi_{m,\alpha }^{2}\;\mathrm{without}\;\mathrm{abnormal}\;\mathrm{observation}\\ H_{1}:\gamma_{k}^{}>\chi_{m,\alpha }^{2}\;\;\mathrm{with}\;\mathrm{abnormal}\;\mathrm{observation} \end{array}\right.$$

where the threshold $\chi_{m,\alpha }^{2}$ can be achieved from the $\chi_{}^{2}$ distribution table.

Upon the identification of abnormal observations, we further develop a novel robust CKF with scaling factor based on the Mahalanobis distance criterion to improve the performance of vehicular INS/GNSS integration by resisting the influence of abnormal observations on system state estimate.

**Remark** **1.** *In (35), if* α *is chosen as a very small value, the judging index* $\gamma_{k}^{}$ *will be smaller than* $\chi_{m,\alpha }^{2}$ *. This means the system works under the normal condition, i.e., the nonlinear system described by (16) does not involve any abnormal observation. However, if* ${\tilde{z}}_{k}$ *and* $P_{{\hat{z}}_{k/k-1}}$ *do not meet the condition given by (35), it can be concluded with high probability* (1−α) *that there are violations to the “a priori” assumption, e.g., there may be an unknown abnormal observation involved in the observation model.*

#### 3.2.2. Determination of the Robust Factor Based on the Mahalanobis Distance Criterion

In this paper, a novel robust CKF with scaling factor is developed to address the influence of abnormal observations on system state estimate. If the judging index $\gamma_{k}^{}$ is not greater than $\chi_{m,\alpha }^{2}$, the standard CKF will be carried out, otherwise a scaling factor $\kappa_{k}$, which is also called the robust factor, will be introduced to inflate the observation noise covariance R. Accordingly, the predicted innovation covariance for the proposed robust CKF should be changed to:

(37) $$P_{{}_{{\tilde{z}}_{k/k-1}}}^{*}=H_{k}P_{k/k-1}H_{k}^{T}+\kappa_{k}R$$

Substituting (37) into (33) and making (35) hold, the following equation can be obtained:

(38) $$g(\kappa_{k})=\gamma_{k}^{*}-\chi_{m,\alpha }^{2}={\tilde{z}}_{k}^{T}{\left(P_{{\tilde{z}}_{k/k-1}^{}}^{*}\right)}^{-1}{\tilde{z}}_{k}^{}-\chi_{m,\alpha }^{2}=0$$

It can be seen from (38) that the determination of the robust factor $\kappa_{k}$ is a problem of solving the nonlinear equation, and it can be solved iteratively by using Newton’s method [16,39]:

(39) $$\kappa_{k}(i+1)=\kappa_{k}(i)-\frac{g[\kappa_{k}(i)]}{g^{\prime }[\kappa_{k}(i)]}$$

where *i* represents the *i-*th iteration. Substituting (38) into (39), we have:

(40) $$\kappa_{k}(i+1)=\kappa_{k}(i)-\frac{{\tilde{z}}_{k}^{T}{\left(P_{{\tilde{z}}_{k/k-1}^{}}^{*}\right)}^{-1}{\tilde{z}}_{k}^{}-\chi_{m,\alpha }^{2}}{{\tilde{z}}_{k}^{T}{\left[{\left(P_{{\tilde{z}}_{k/k-1}^{}}^{*}\right)}^{-1}\right]}^{\prime }{\tilde{z}}_{k}^{}}$$

According to the following derivative formula for the inverse matrix [39]:

(41) $$\frac{d}{dt}(A^{-1})=-A^{-1}\frac{dA}{dt}A^{-1}$$

it can be easily achieved that:

(42) $$\kappa_{k}(i+1)=\kappa_{k}(i)+\frac{{\tilde{z}}_{k}^{T}{\left(P_{{\tilde{z}}_{k/k-1}^{}}^{*}(i)\right)}^{-1}{\tilde{z}}_{k}^{}-\chi_{m,\alpha }^{2}}{{\tilde{z}}_{k}^{T}\left[{\left(P_{{\tilde{z}}_{k/k-1}^{}}^{*}(i)\right)}^{-1}R{\left(P_{{\tilde{z}}_{k/k-1}^{}}^{*}(i)\right)}^{-1}\right]{\tilde{z}}_{k}^{}}\;(i=0,1,2,3,\cdots )$$

where ***A*** in (41) is an invertible matrix as a function of *t*.

The above iterative process to determine the robust factor $\kappa_{k}$ is initialized as 1, e.g., $\kappa_{k}(0)=1$, and it will be terminated when the judging index satisfies $\gamma_{k}^{*}(i)\leq \chi_{m,\alpha }^{2}$, or the iteration index is greater than the predetermined threshold.

**Remark** **2.** *If* $\gamma_{k}^{*}\leq \chi_{m,\alpha }^{2}$ *, **R** will remain unchanged, i.e.,* $\kappa_{k}=1$ *. For the proposed robust CKF, when* $\gamma_{k}^{*}>\chi_{m,\alpha }^{2}$ *, because of* $\kappa_{k}$ *, **R** will be “enlarged”. Accordingly, the actual observation will be less weighted, while the predicted state will be more weighted in the observation update procedure of CKF.*

**Remark** **3.** *In practical applications, a simplified way to determine the robust factor can also be considered to improve the computational efficiency. An inflated observation noise covariance results in an inflated innovation vector covariance, thus the robust factor can be directly used to adjust the innovation vector covariance for the filtering robustness, i.e.,:*

(43) $$P_{{}_{{\hat{z}}_{k/k-1}}}^{*}=\kappa_{k}P_{{}_{{\hat{z}}_{k/k-1}}}^{}$$

In this case, the robust factor $\kappa_{k}$ can be solved analytically rather than iteratively as in (42). It is directly calculated as:

(44) $$\kappa_{k}=\frac{\gamma_{k}}{\chi_{\alpha ,m}^{2}}$$

**Remark** **4.** *The judging index* $\gamma_{k}^{*}$ *and the robust factor* $\kappa_{k}$ *in the proposed robust CKF are computed using the information at the current epoch only. For many other methods, the robust factors are determined via both current and historical information. Thus, the proposed method has a smaller computational load and is more sensitive to the abnormal observation at the present epoch, leading to the effectiveness in responding to dynamically changing abnormal observations.*

#### 3.2.3. Proposed Robust Cubature Kalman Filter

**Figure 1:**
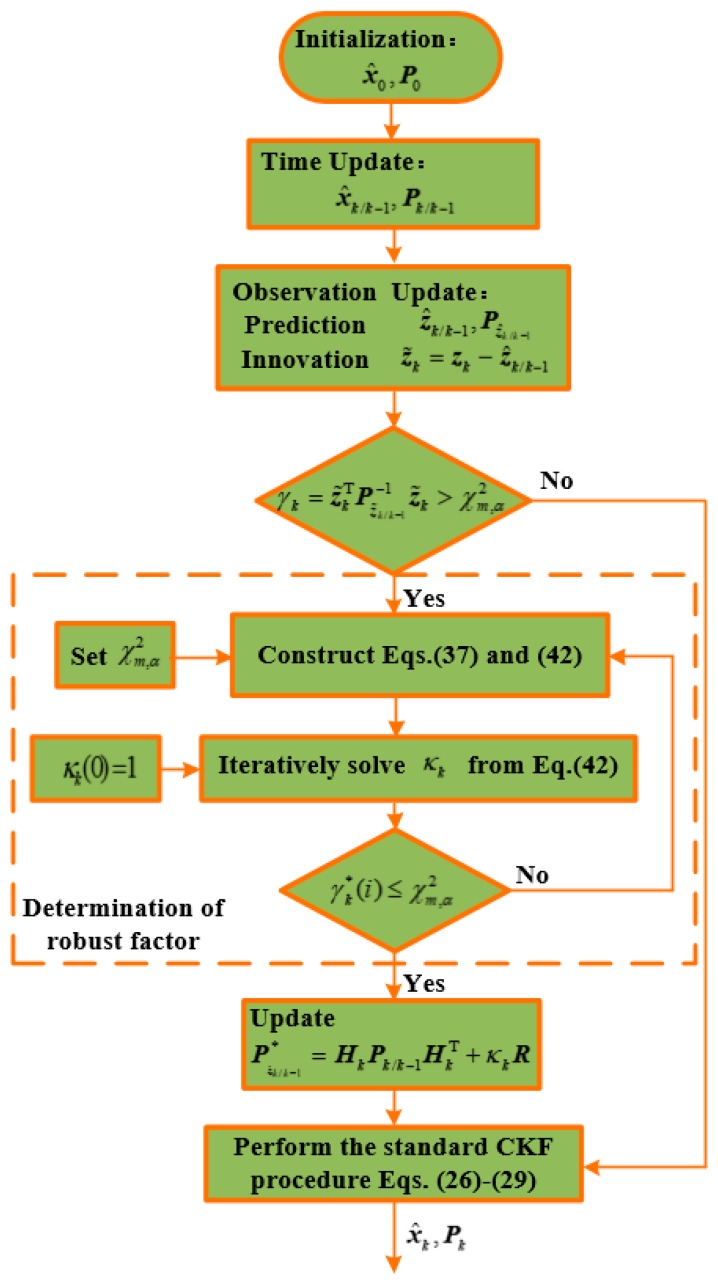
The procedure of the proposed robust CKF based on Mahalanobis Distance Criterion.

For the vehicular INS/GNSS integrated system, if the judging index $\gamma_{k}^{}$ is not greater than $\chi_{m,\alpha }^{2}$ the standard CKF will be carried out, otherwise the robust factor $\kappa_{k}$ will be introduced to inflate the observation noise covariance R. The procedure of the proposed robust CKF is shown in Figure 1, which can be summarized as:

*Step 1:* Initialization.

Initiate the filter with ${\hat{x}}_{0}$ and $P_{0}$ as (17).

*Step 2:* Time Update.

Calculate the state prediction ${\hat{x}}_{k/k-1}$ and its covariance $P_{k/k-1}$ through (22) and (23).

*Step 3:* Observation Update. 

(i) Compute the innovation vector ${\tilde{z}}_{k}$ and its covariance $P_{{\hat{z}}_{k/k-1}}$;

(ii) Set $\kappa_{k}(0)=1$ and obtain the judging index $\gamma_{k}^{*}$;

(iii) If $\gamma_{k}^{*}\leq \chi_{m,\alpha }^{2}$,

- Perform the standard CKF procedure (26)–(29) to obtain the system state estimate.Else,
- Determine the robust factor $\kappa_{k}$ by iteratively solving (42) until the judging index satisfies $\gamma_{k}^{*}(i)\leq \chi_{m,\alpha }^{2}$.
- Change the innovation covariance as (37).
- Execute the standard CKF procedure (26)–(29) to update the system state estimate.

*Step 4:* Repeat Steps 2 and 3 for the process of all the samples.

## 4. Performance Evaluation and Analysis

Simulations and practical experiments were conducted to comprehensively evaluate the proposed Mahalanobis Distance Criterion-based robust CKF (MDC-RCKF) for vehicular INS/GNSS integration. Comparison analysis of the proposed MDC-RUKF with the standard CKF, and H-infinity strategy based robust CKF (HI-RCKF) in [29] was also conducted to demonstrate the performance improvement.

### 4.1. Simulations and Analysis

Monte Carlo simulations were carried out for the performance evaluation of the proposed MDC-RCKF in vehicular INS/GNSS integration with abnormal observations. The two typical cases of abnormal observations, i.e., outliers in observation and contaminated Gaussian noise distribution, are considered in this section.

**Figure 2:**
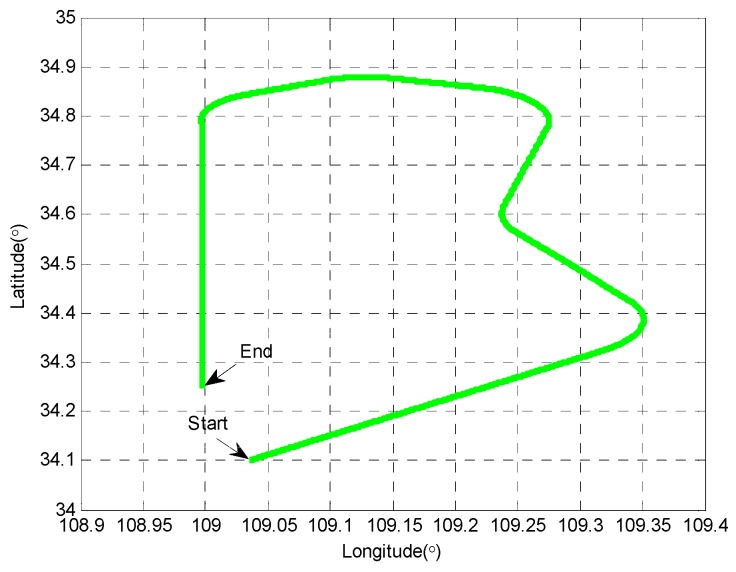
The movement trajectory of the vehicle.

The simulated movement trajectory for the vehicle is shown in Figure 2, which includes various maneuvers such as uniform linear motion, accelerated linear motion, turning and so on. The simulation parameters are listed in Table 1. The simulation time lasts 1000 s and the filtering period is 1 s. To identify abnormal observations, $\chi_{m,\alpha }^{2}$ for the proposed MDC-RCKF was chosen as 12.592, which was derived from the $\chi^{2}$ distribution with the confidence level of 95% (α=0.05) and 6 degrees of freedom (*m* = 6). Monte Carlo simulations were conducted at the same conditions to compare the performance of the proposed MDC-RCKF with the standard CKF and HI-RCKF. The root mean squared errors (RMSE) is adopted to evaluate the navigation performance of the proposed MDC-RCKF for INS/GNSS integration. It is defined as:

(45) $$RMSE_{k}=\sqrt{\frac{1}{M}\sum_{i=1}^{M}({\hat{x}}_{k}^{i}-x_{k}^{true}{)}^{2}}$$

where *M* is the Monte Carlo runs, which is set as 100 for this simulation.

#### 4.1.1. Navigation Accuracy Evaluation

In order to evaluate the performance of the proposed MDC-RCKF in terms of abnormal observations, the following two typical cases are considered in the simulation analysis:

(i) Outliers in observation: There exist outliers in the observation of the INS/GNSS integrated navigation system. In the simulation, the horizontal position error of 15 m was artificially added into the observation described by (15) every 200 s.
(ii) Contaminated Gaussian noise distribution. The nominal Gaussian distribution of the observation noise in INS/GNSS integration is contaminated by another Gaussian distribution, i.e.,:
  (46) $$\rho_{\mathrm{actual}}=(1-\varepsilon )\rho_{\mathrm{nominal}}+\varepsilon \rho_{perturbing}$$
  where ε is the ratio of the perturbing distribution, which is selected as 0<ε≤0.5. The $\rho_{perturbing}$ obeys the Gaussian distribution with a larger standard deviation. In this section, the standard deviation of the perturbing distribution is assumed to be 5 times larger than that of the nominal distribution, and ε is set to 0.2.

(A) Outliers in Observation

**Figure 3:**
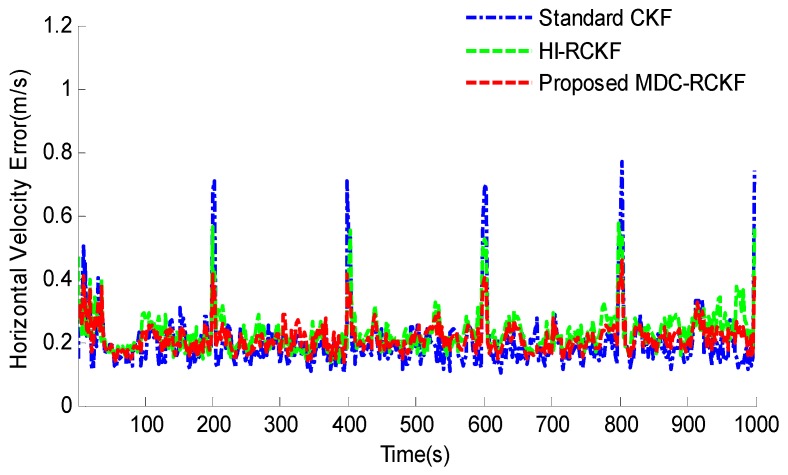
RMSEs of horizontal velocity for the vehicular INS/GNSS integration in the presence of outliers in observation.

**Figure 4:**
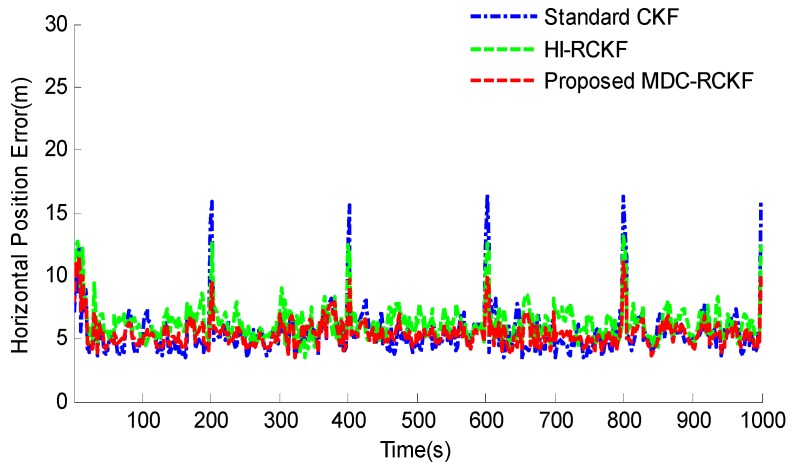
RMSEs of horizontal position for the vehicular INS/GNSS integration in the presence of outliers in observation.

Figure 3; Figure 4 show the RMSEs of both horizontal velocity and position obtained by the standard CKF, HI-RCKF and proposed MDC-RCKF, respectively. For the time periods without outliers in observation, all the above three filters can accurately estimate both horizontal velocity and position for the vehicle. 

However, the navigation accuracy achieved by HI-RCKF is slightly lower that of the standard CKF and proposed MDC-RCKF. This is because HI-RCKF has no identification process for abnormal observations, i.e., when the observation is accurate, HI-RCKF can only obtain the suboptimal results for the navigation solution.

Further, for the time points of 200 s, 400 s, 600 s, 800 s and 1000 s with outliers in observation, the navigation accuracy of the standard CKF severely declines due to the influence of abnormal observations. HI-RCKF can weaken the outliers in observation to some extent by using the H-infinity strategy. The achieved mean RMSEs in both horizontal velocity and position are 23.4% and 20% smaller than those of the standard CKF, respectively. However, this method still has pronounced errors in the navigation solution. In contrast, the proposed MDC-RCKF achieves the highest navigation accuracy for these time points by adjusting the robust factor calculated from Mahalanobis distance criterion. Its mean RMSEs in both horizontal velocity and position are at least 13% and 23.7% smaller than those of HI-RCKF, respectively.

**Figure 5:**
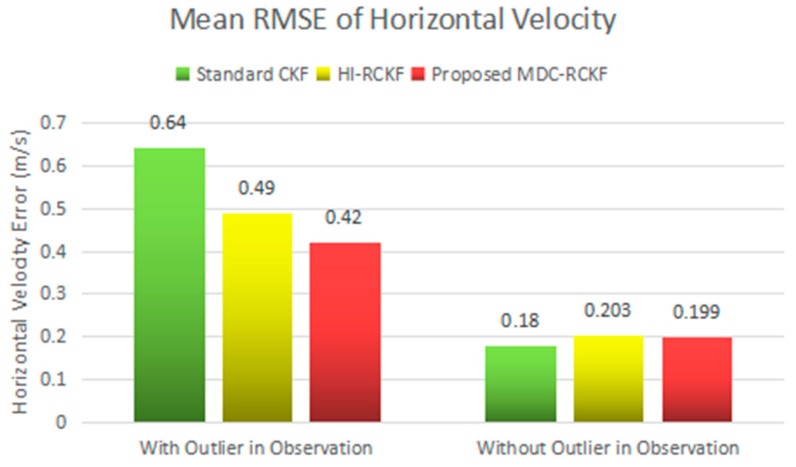
Mean RMSEs of horizontal velocity for the vehicular INS/GNSS integration in the presence of outliers in observation.

**Figure 6:**
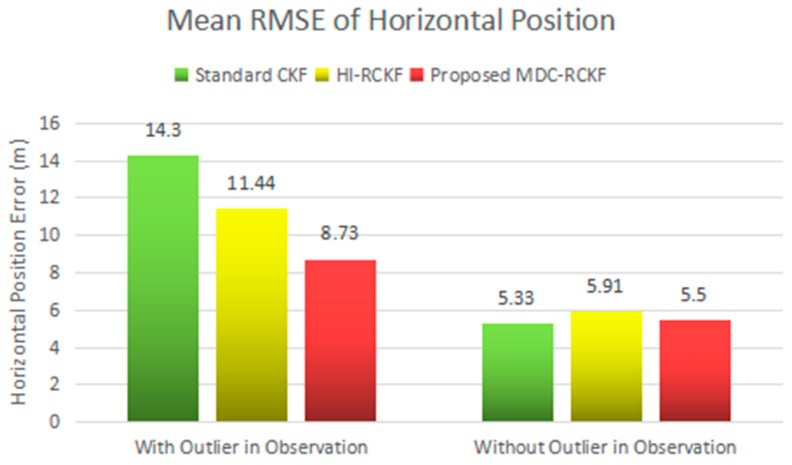
Mean RMSEs of horizontal position for the vehicular INS/GNSS integration in the presence of outliers in observation.

The intuitive comparison of the standard CKF, HI-RCKF and proposed MDC-RCKF for the mean RMSEs of both horizontal velocity and position are described in Figure 5 and Figure 6. The relevant results also illustrate that the proposed MDC-RCKF has better robustness to inhibit the outliers in observation comparing to the other filters. Thus, the proposed method can obtain a superior performance for vehicular INS/GNSS integration.

(B) Contaminated Gaussian Noise Distribution

**Figure 7:**
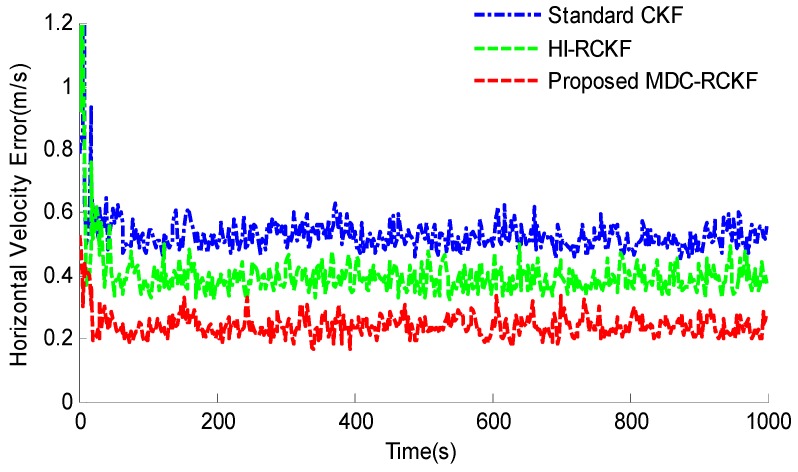
RMSEs of horizontal velocity for the vehicular INS/GNSS integration in the presence of contaminated Gaussian noise distribution.

**Figure 8:**
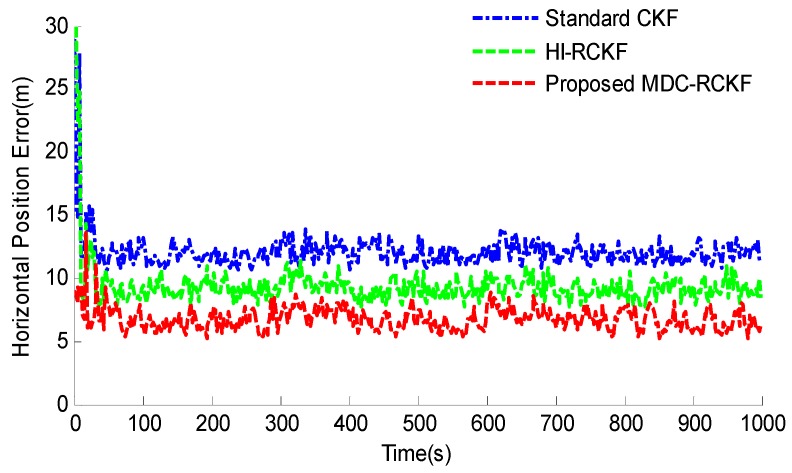
RMSEs of horizontal position for the vehicular INS/GNSS integration in the presence of contaminated Gaussian noise distribution.

**Figure 9:**
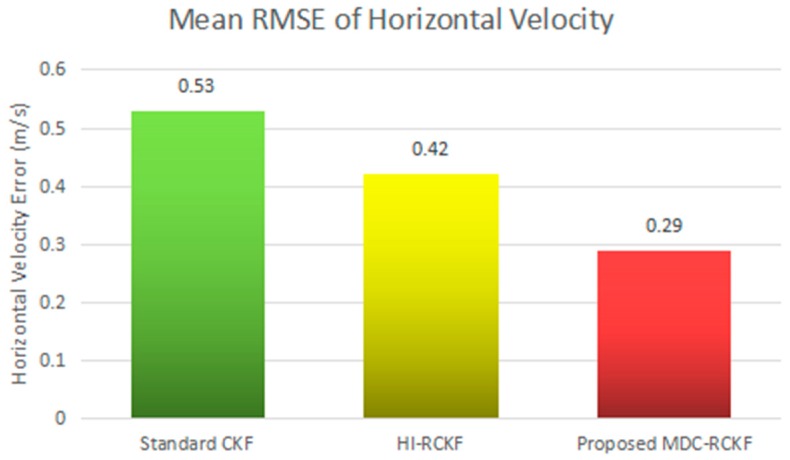
Mean RMSEs of horizontal velocity for the vehicular INS/GNSS integration in the presence of contaminated Gaussian noise distribution.

**Figure 10:**
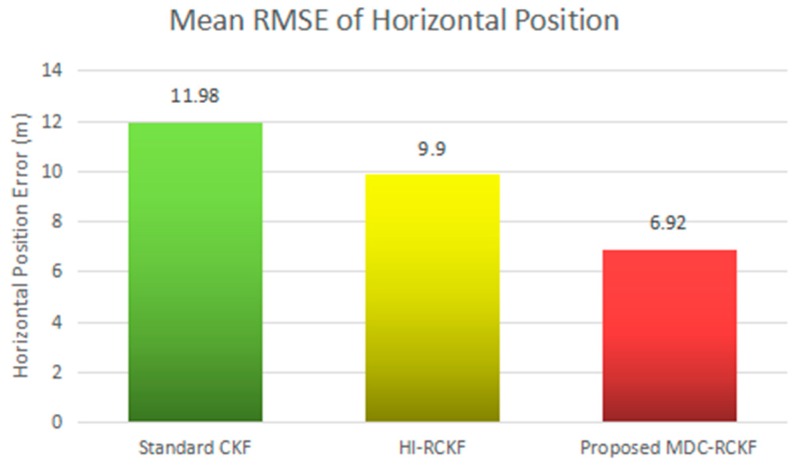
Mean RMSEs of horizontal position for the vehicular INS/GNSS integration in the presence of contaminated Gaussian noise distribution.

Figure 7 and Figure 8 depict the RMSEs of both horizontal velocity and position calculated by the standard CKF, HI-RCKF and proposed MDC-RCKF for the vehicular INS/GNSS integration with contaminated Gaussian noise distribution. The mean RMSEs of horizontal velocity and position obtained by the standard CKF, HI-RCKF and proposed MDC-RCKF in this case are also intuitively compared and shown in Figure 9 and Figure 10. Figure 7 Figure 8 Figure 9 Figure 10 illustrate a similar phenomenon as the case of outliers in observation. When the observation noise is perturbed by another Gaussian distribution, the horizontal velocity and position errors obtained by the standard CKF are largest among the above three filters, and its mean RMSEs in horizontal velocity and position are 0.53 m/s and 11.98 m, respectively. The accuracy of HI-RCKF is relatively superior to that of the standard CKF due to the ability to inhibit the contaminated observation noise distribution. The obtained mean RMSEs in horizontal velocity and position are 0.42 m/s and 9.90 m, which are 10.9% and 17.4% smaller than those of the standard CKF. As expected, the proposed MDC-RCKF has higher navigation accuracy compared to the standard CKF and HI-RCKF, leading to the mean RMSEs in horizontal velocity and position at 0.29 m/s and 6.92 m, which are 32% and 30.1% smaller than those of HI-RCKF, respectively.

#### 4.1.2. Computational Performance Evaluation

Simulation trials were also conducted to investigate the computational performance of the proposed MDC-RCKF for the above two typical cases of abnormal observations. The Monte Carlo simulation trials were conducted with Matlab programs on an Intel(R) Core(TM) i5-9400F 2.9 GHz PC with 8 GB memory. The average computational times (i.e., the filtering times for each Monte Carlo run) of the standard CKF, HI-RCKF and proposed MDC-RCKF are shown in Table 2. In order to eliminate the effect resulted from the difference of computers, the relative efficiencies of HI-RCKF and MDC-RCKF in comparison with the standard CKF are also calculated and listed in Table 2. It can be seen from Table 2 that the standard CKF has the smallest computational time among the above three filtering methods. The computational time of HI-RCKF is at least 198.28% larger than that of the standard CKF. This is because the use of the H-infinity strategy to resist the influence of abnormal observations involves a large computational load at each iteration during the HI-RCKF filtering procedure. In contrast, the proposed MDC-RCKF shows a superior computational performance than HI-RCKF. For the case with outliers in observation, due to the use of the identification process of abnormal observations, the computational time of MDC-RCKF is 55.83% smaller than that of HI-RCKF. For the case with contaminated Gaussian noise distribution, the computational time of MDC-RCKF is also 22.14% smaller than that of HI-RCKF, even though the determination of the robust factor is involved in each iteration during the MDC-RCKF filtering procedure. Moreover, the MDC-RCKF computational time is only slightly greater than the standard CKF one due to the determination procedure of the robust factor. The above analysis demonstrates that the proposed MDC-RCKF has a faster computation speed than HI-RCKF, which is sufficient to achieve the real-time performance for vehicular INS/GNSS integration.

The above simulations and analysis for navigation accuracy and computational performance verify that the proposed MDC-RCKF can effectively identify abnormal observations and further inhibit their influence on the navigation solution, leading to smaller navigation error than the standard CKF and HI-RCKF for vehicular INS/GNSS integration. In addition, the proposed MDC-RCKF also has a better computational performance than HI-RCKF and is sufficient to achieve the real-time performance for vehicular INS/GNSS integration.

### 4.2. Practical Experiment and Analysis

To further evaluate the performance of the proposed MDC-RCKF, an experiment of terrestrial vehicular navigation was carried out and the comparison with standard CKF and HI-RCKF was also conducted.

#### 4.2.1. Experiment Setup

The experimental system of terrestrial vehicular navigation is made up of an ampere-voltage meter, a battery, a controller and a self-made INS/GNSS integration navigation system. This navigation system consists of a NV-IMU300 inertial measurement unit, and a JAVAD Lexon-GGD112T GPS receiver. The constant drift of the gyro is 0.06 ${}^{\circ }/h$; the accelerometer bias is $5\times 10^{-3}\;g$; the sampling frequency for IMU measurements is 25 Hz; the GNSS’s horizontal positioning accuracy, altitude accuracy and velocity accuracy are 5 m, 8 m and 0.05 m/s, respectively. Moreover, another JAVAD Lexon-GGD112T GPS receiver placed at a local reference station was utilized along with the one mounted on the vehicle to provide the differential GPS (DGPS) data. Because DGPS can provide the high accuracy positioning information (≤0.1 m) via post processing, the DGPS data are used as the reference for the comparison with the filtering results of the INS/GNSS integration.

#### 4.2.2. Experimental Process

**Figure 11:**
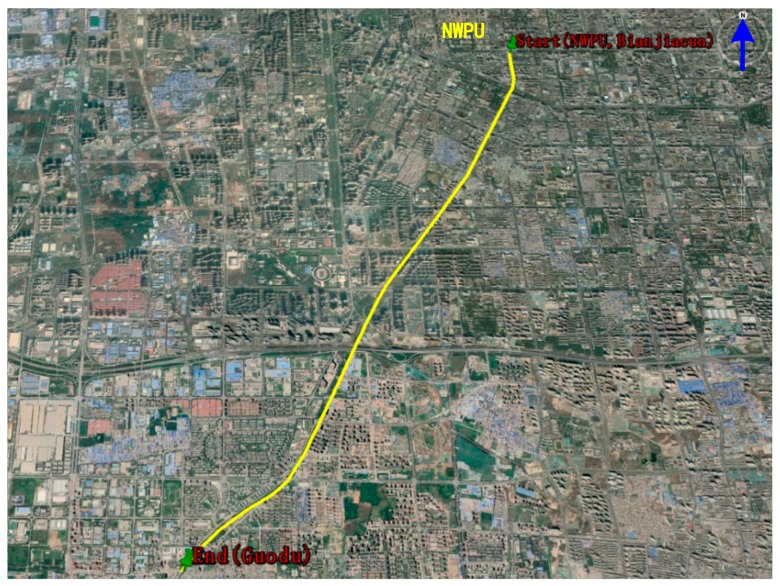
The movement trajectory of the experimental vehicle.

The experiment of terrestrial vehicular navigation was conducted near the Youyi Campus of Northwestern Polytechnical University (NWPU) at the city of Xi’an, Shaanxi, China. The start point of the experimental vehicle was at North latitude $34.243^{\circ }$, East longitude $108.919^{\circ }$ and Altitude 412 m. After the initialization of the INS/GNSS integrated navigation system for 5 min, the experimental vehicle started to move towards the Southwestern direction at the velocity of 5 m/s and terminated at the Guodu station (at North latitude $34.162^{\circ }$, East longitude $108.861^{\circ }$, Altitude 429 m). The movement trajectory of the experimental vehicle is depicted in Figure 11. The vehicle travelling time is about 2000 s, and the vehicle travelling distance is about 11 km with the average velocity of 20 km/h.

#### 4.2.3. Experimental Results and Analysis

Practical experimental data was processed under the same conditions by the standard CKF, HI-RCKF and proposed MDC-RCKF, respectively. $\chi_{m,\alpha }^{2}$ in the proposed MDC-RCKF was also chosen as 12.592 for identification of abnormal observations. The other initial parameters were set to the same values as those in the simulation analysis.

**Figure 12:**
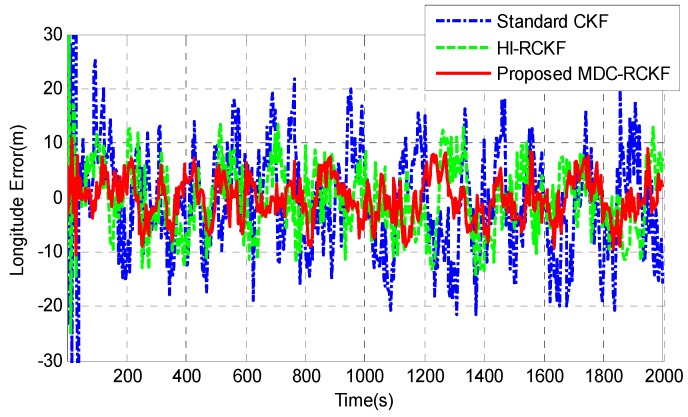
Longitude errors for the vehicular INS/GNSS integration in practical experiment case.

**Figure 13:**
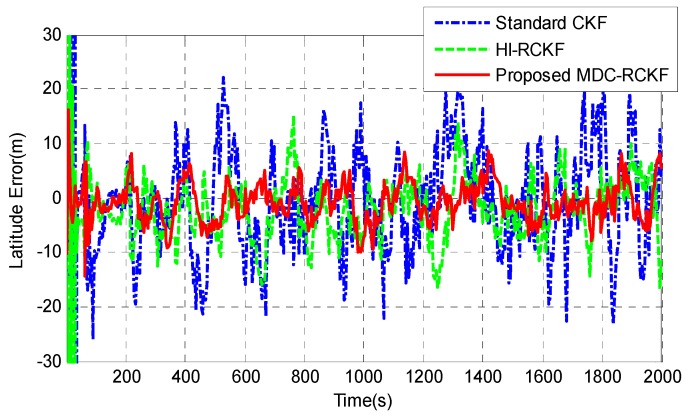
Latitude errors for the vehicular INS/GNSS integration in practical experiment case.

Figure 12; Figure 13 give the position errors in longitude and latitude of the vehicle by the standard CKF, HI-RCKF and proposed MDC-RCKF. During the test process, the GNSS signals were shielded by the tall buildings or tree canopies in the environment, leading to the outliers in observation. As shown in Figure 12; Figure 13, the solution of the standard CKF is severely disturbed by the abnormal observations in the environment, leading to the largest position errors in longitude and latitude, which are within (−21.57 m, 21.77 m) and (−23.02 m, 22.14 m). HI-RCKF has better navigation accuracy than the standard CKF, leading to the position errors in longitude and latitude within (−14.45 m, 13.53 m) and (−16.73 m, 15.02 m). However, obvious oscillations still exist in the longitude and latitude error curves of HI-RCKF. Compared to the standard CKF and HI-RCKF, the position errors in longitude and latitude obtained by the proposed MDC-RCKF are much smaller, which are within (−9.42 m, 8.74 m) and (−10.04 m, 8.89 m), respectively.

**Figure 14:**
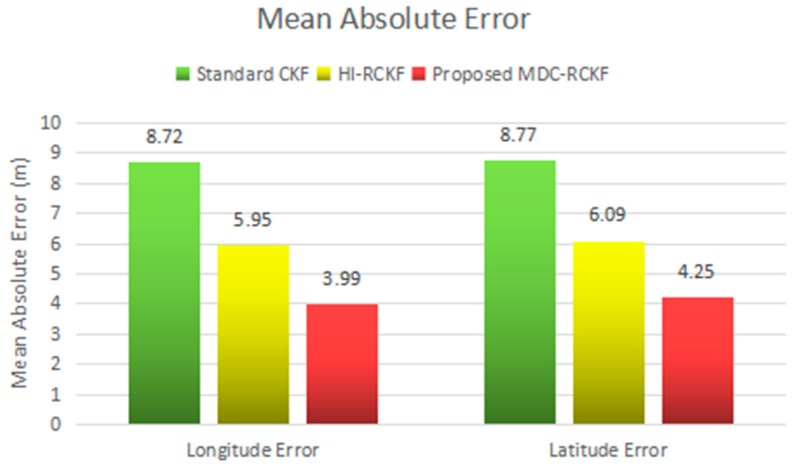
MAEs in longitude and latitude for the vehicular INS/GNSS integration in the experimental case.

**Figure 15:**
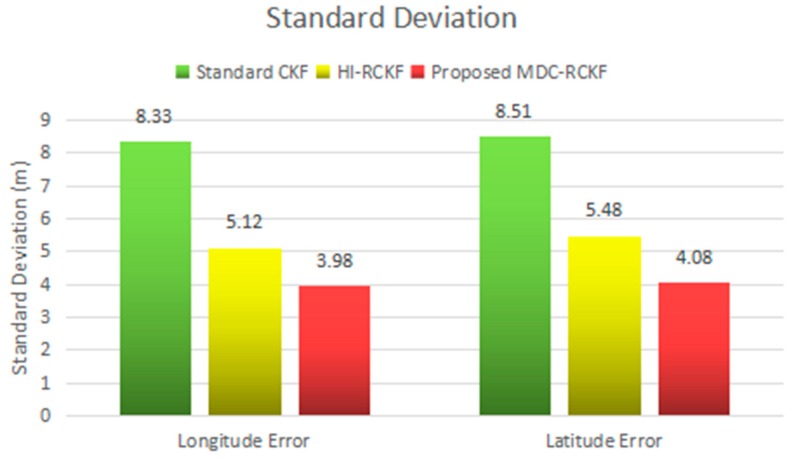
STDs of position error in and longitude latitude for the vehicular INS/GNSS integration in the experimental case.

The mean absolute errors (MAE) and the standard deviations (STD) of the position errors in longitude and latitude for the comparison of the standard CKF, HI-RCKF and proposed MDC-RCKF are intuitively described in Figure 14; Figure 15. These results also verify that the proposed MDC-RCKF is capable of enhancing the filtering robustness and outperforms the standard CKF and HI-RCKF in terms of positioning accuracy in the presence of abnormal observations for the vehicular INS/GNSS integration.

## 5. Conclusions

This paper presents a novel robust CKF with scaling factor for nonlinear INS/GNSS integration. The contribution of this paper is that novel theories of the Mahalanobis distance are established for identification of abnormal observations and further for improvement of the robustness of the standard CKF. The abnormal observations involved in nonlinear INS/GNSS integration are identified using the Mahalanobis distance criterion. Subsequently, a robust factor (scaling factor) is introduced into the standard CKF to inflate the observation noise covariance and further decreases the filtering gain to resist the influence of abnormal observations on navigation solution. The simulation and practical experimental results as well as comparison analysis demonstrate that the proposed robust CKF has a strong robustness against abnormal observations, resulting in enhanced performance for INS/GNSS integrated vehicle navigation.

Future research work will focus on the improvement of the proposed robust CKF. The proposed robust CKF will be combined with advanced artificial intelligence technologies such as machine learning, deep learning and neural network to automatically identify abnormal observations and resist their influence on navigation solution.

## Figures and Tables

**Table 1:** Simulation parameters.

Parameters			Values
Initial Parameters	Velocity (east-north-up)		(5 m/s, 3 m/s, 0 m/s)
	Position (longitude-latitude-altitude)		($109.385^{\circ },34.100^{\circ },400\;m$)
Initial Errors	Attitude (pitch-roll-yaw)		$\left(1^{\prime },1^{\prime },1.5^{\prime }\right)$
	Velocity (east-north-up)		(0.3 m/s, 0.3 m/s, 0.3 m/s)
	Position (longitude-latitude-altitude)		(8 m, 8 m, 12 m)
INS	Gyro	Constant drift	0.1 °/h
		Random walk coefficient	0.01${}^{\circ}/\sqrt{h}$
		Sampling period	0.05 s
	Accelerometer	Zero-bias	1 × 10^−3^ g
		Random walk coefficient	$10^{-4}\;g\cdot \sqrt{s}$
		Sampling period	0.05 s
GNSS	Velocity Accuracy (RMS)		0.05 m/s
	Horizontal Positioning Accuracy (RMS)		3 m
	Altitude Accuracy (RMS)		5 m
	Data update rate		1 s

**Table 2:** The average computational times and relative efficiencies of the standard CKF, HI-RCKF and proposed MDC-RCKF for the simulation cases.

Filtering Methods	Outliers in Observation Case		Contaminated Gaussian Noise Distribution Case	
	Average Computational Time (s)	Relative Efficiency	Average Computational Times (s)	Relative Efficiency
Standard CKF	1.6287	1	1.6132	1
HI-RCKF	4.8582	2.9828	4.9643	3.0773
Proposed MDC-RCKF	2.1455	1.3173	3.8653	2.3960
